# Knockdown of myeloid cell hypoxia-inducible factor-1α ameliorates the acute pathology in DSS-induced colitis

**DOI:** 10.1371/journal.pone.0190074

**Published:** 2017-12-20

**Authors:** Veronika Bäcker, Fung-Yi Cheung, Jens T. Siveke, Joachim Fandrey, Sandra Winning

**Affiliations:** 1 Institut für Physiologie, Universität Duisburg-Essen, Essen, Germany; 2 Division of Solid Tumor Translational Oncology, German Cancer Consortium (DKTK), partner site Essen, University Hospital Essen, Essen, Germany; University College Dublin, IRELAND

## Abstract

Inflammation and hypoxia are hallmarks of inflammatory bowel disease. Low oxygen levels activate hypoxia-inducible factors as central transcriptional regulators of cellular responses to hypoxia, particularly in myeloid cells where hypoxia-inducible factors control immune cell function and survival. Still, the role of myeloid hypoxia-inducible factor-1 during inflammatory bowel disease remains poorly defined. We therefore investigated the role of hypoxia-inducible factor-1 for myeloid cell function and immune response during colitis. Experimental colitis was induced by administration of 2.5% dextran sulfate sodium to mice with a conditional knockout of hypoxia-inducible factor-1α in myeloid cells and their wild type siblings. Murine colon tissue was examined by histologic analysis, immunohistochemistry, and quantitative polymerase chain reaction. Induction of experimental colitis increased levels of hypoxia and accumulation of hypoxia-inducible factor-1α positive cells in colon tissue of both treated groups. Myeloid hypoxia-inducible factor-1α knockout reduced weight loss and disease activity index when compared to wild type mice. Knockout mice displayed less infiltration of macrophages into intestinal mucosa and reduced mRNA expression of markers for dendritic cells and interleukin-17 secreting T helper cells. Expression of inflammatory and anti-inflammatory cytokines also showed a reduced and delayed induction in myeloid hypoxia-inducible factor-1α knockout mice. Our results show a disease promoting role of myeloid hypoxia-inducible factor-1 during intestinal inflammation. This might result from a hypoxia-inducible factor-1 dependent increase in pro-inflammatory interleukin-17 secreting T helper cells in the absence of obvious changes in regulatory T cells. In contrast, knockout mice appear to shift the balance to anti-inflammatory signals and cells resulting in milder intestinal inflammation.

## Introduction

Inflammatory bowel disease (IBD) in humans has two major forms: Crohn’s disease and ulcerative colitis. Both inflammatory disorders are associated with dysregulated innate and adaptive immune response [1] and characterized by hypoxia [2]. In most healthy tissues O_2_ tension varies from 15 mmHg to 50 mmHg. Inflamed or diseased tissues can reach O_2_ levels below 5 mmHg due to vascular damage, intensive metabolic activity of bacteria and other pathogens, and large numbers of infiltrating cells [3]. Cells shift their metabolism from aerobic oxidative phosphorylation to anaerobic glycolysis, stimulate the production of vasorelaxants and generate new blood vessels to adapt to hypoxia. Hypoxic adaptation is regulated by transcription factors, called hypoxia-inducible factors (HIFs) [3]. HIFs are heterodimeric complexes composed of a hypoxia inducible α-subunit and a constitutively expressed β-subunit. Under normoxic conditions, HIF-α proteins are rapidly degraded by the ubiquitin-proteasome pathway after hydroxylation by oxygen-dependent prolyl hydroxylases (PHD). Additionally, HIF-α transcriptional activity is repressed by factor inhibiting HIF (FIH) which blocks the recruitment of coactivators by O_2_ dependent asparagine hydroxylation. Hypoxia allows HIF-α accumulation, nuclear translocation and dimerization with β-subunits, recruitment of additional transcriptional coactivators, and finally binding to hypoxia-response elements and transcription of target genes [4]. Beside hypoxia, inflammation leads to activation of HIF through the nuclear factor-kappa B [5]. HIF-1α is expressed in nearly every immune cell of either the adaptive or the innate immune system [6–8]. In myeloid cells, HIF-1 was shown to be essential for aggregation, motility, invasiveness, and bacterial killing [6]. In addition, HIF-1 has been revealed to have a protective role in various immune cells and epithelial cells during IBD [9–11]. Mice with a knockout of HIF-1α in dendritic cells (DCs) exhibited more severe intestinal inflammation and impaired induction of regulatory T cells when compared to mice with functional dendritic HIF-1α [9]. Cell specific deletion of HIF-1α in T cells led to a more severe colonic inflammation with up-regulation of Th1 and Th17 cells [10]. In colonic epithelial cells HIF-1 is a protective element in inflammatory mucosal disease with barrier-protective gene products transcriptionally regulated by HIF-1 [11]. The role of myeloid HIF-1 during IBD still remains unknown. Cramer et al. could show that myeloid HIF-1α essentially drives immune cell functions. The authors pointed out that HIF-1α lacking myeloid cells amongst others fail to upregulate glycolytic metabolism under inflammatory conditions [6]. More recently Flück et al. showed that HIF-1α plays a crucial role for dendritic cell function during intestinal inflammation indicating that HIF-1, even in a small subset of immune cells, may determine the outcome of a disease [9].

Thus, we hypothesize that HIF-1 plays an important role for myeloid cell function during intestinal inflammation. We provide first evidence for a disease promoting role of myeloid HIF-1 during colitis. Loss of myeloid HIF-1α resulted in a milder DSS-induced colitis with lower numbers of infiltrating immune cells. These results underline the pivotal role of HIF-1α during inflammation of the colon in an immune cell specific context.

## Materials and methods

### Animals

Female C57BL/6J mice (aged 10–12 weeks) with a cell-specific knockout of HIF-1α in myeloid cells (Lyz2-Cre/HIF-1α^+f/+f^) and their HIF-1α expressing siblings (HIF-1α^+f/+f^) were used for experiments. To achieve myeloid-specific recombination we crossed mice carrying the HIF-1α conditional allele to mice that expressed Cre-recombinase under control of *lysozyme M* regulatory elements [12]. Mice were purchased from The Jackson Laboratory (Bar Harbor, ME, USA). Genotyping was performed by amplification of genomic DNA extracted from ear punches. The animals were kept under specific pathogen-free conditions in disposable cages with filter tops in a 12-hour light/dark cycle. Sterile drinking water and standard rodent pellets were provided ad libitum. Animals demonstrated physiological habitus and bred regularly. They were kept and treated in accordance with the German law for animal welfare and institutional regulations for animal handling. Experimental procedures were approved by the Landesamt für Natur, Umwelt und Verbraucherschutz Nordrhein-Westfalen (LANUV NRW, reference number 84–02.04.2011.A098).

### Induction and monitoring of DSS-induced colitis

Acute colonic inflammation was induced by oral administration of 2.5% (w/v) dextran sulfate sodium (DSS) (MP Biomedicals, MW 36–50 kDa) in drinking water for four to six days. DSS solution was renewed after three days [13,14]. Animal weight loss, stool consistency, and fecal blood were recorded daily for each animal. These parameters were used to calculate an average Disease Activity Index (DAI) for each animal as described by Cooper et al. [15]. The maximum DAI score was 12 based on a scoring system of 1–4 for each parameter (weight loss + stool consistency + occurrence of fecal blood): score of 0, no weight loss, normal stool, no blood; score of 1, 1%-5% weight loss; score of 2, 5%-10% weight loss, loose stool, positive hema screen test (Immunostics.Inc.); score of 3, 10%-20% weight loss; and score of 4, more than 20% weight loss, diarrhea, and blood visible in stool.

### Cell culture

Bone-marrow derived macrophages from wild type and knockout mice were isolated from femurs. After 24 h of cultivation, non-adherent monocytes were harvested and cultivated for 7 days under addition of L-cell-conditioned MEM-Medium. Hypoxic stimulation (Baker Ruskin Invivo_2_, 1% O_2_/ 5% CO_2_/ 94% N_2_) was performed at day 7 of cultivation for 6 h. To obtain M1 macrophages, cells were incubated with 10 ng/ml LPS while M2 polarization was obtained by treating cells with 20 ng/ml murine IL-4. Both polarization treatments were carried out for 16 h in MEM-Medium without L cell supernatant during hypoxic incubation. Neutrophils from wild type and knockout mice were isolated from femurs according to a protocol described elsewhere [16]. First steps of RNA isolation were performed under hypoxic conditions.

### Real-time PCR

RNA was extracted from colon tissue using RNeasy^®^ Fibrous Tissue Mini Kit (Qiagen) or from cell culture by the acid guanidinium thiocyanate/phenol/chloroform extraction method [17]. cDNA synthesis was performed using the M-MLV Reverse Transcriptase (Promega) according to the manufacturer’s instructions. For real-time PCR amplification MESA GREEN 2x SYBR reaction mixture (Eurogentec) was used. Except for *inos* amplification (62°C for 1 min), cDNA was amplified by 40 cycles of 95°C for 15 s and 60°C for 1 min with gene specific primers (Invitrogen) (see Table 1). Relative changes in mRNA levels were calculated by the ΔΔCt method, with ribosomal protein S16 as reference standard.

**Table 1:** Primer sequences of specific PCR products for each gene analyzed.

Gene	Sense	Antisense
*cd11c*	GGACGGTGCTGAGTTCGGACACAG	CCACAAGCCAACAGCCAGGAAGG
*f4/80*	TCTGGGGAGCTTACGATGGA	GAATCCCGCAATGATGGCAC
*fizz*	TCCCAGTGAATACTGATGAGA	CCACTCTGGATCTCCCAAGA
*hif-1α exon2*	CATCCAGAAGTTTTCTCACACG	GGCGAAGCAAAGAGTCTGAA
*ifnγ*	GGTCAACAACCCACAGGTCC	CAGCGACTCCTTTTCCGCTT
*il-1β*	CCTCTCCAGCCAAGCTTCCT	TTTGGAAGCAGCCCTTCATC
*il-6*	TCCTACCCCAATTTCCAATGC	CATAACGCACTAGGTTTGCCG
*il-10*	TGCCCCAGGCAGAGAAGCAT	GGGAGAAATCGATGACAGCGCC
*il-17a*	TCATCCCTCAAAGCTCAGCG	TTCATTGCGGTGGAGAGTCC
*inos*	ACATCGACCCGTCCACAG	CAGAGGGGTAGGCTTGTCTC
*lipocalin-2*	ACGGACTACAACCAGTTCGC	CATTGGTCGGTGGGGACAGA
*mcp-1 (BMDM)*	GCTCAGCCAGATGCAGTTAACGCCC	CCTTCTTGGGGTCAGCACAGACCT
*mcp-1 (tissue)*	GTGCTGAAGACCTTAGGGCA	AGCTGTAGTTTTTGTCACCAAGC
*s16*	AGATGATCGAGCCGCGC	GCTACCAGGGCCTTTGAGATGGA
*tnfα*	CGGGGTGATCGGTCCCCAAAG	GGAGGGCGTTGGCGCGCTGG
*ym-1*	GCCAGCAGAAGCTCTCCAGAAGCAA	ACTGAACGGGGCAGGTCCAAACT

### Histology

Tissue sections from distal colon were fixed in 4% paraformaldehyde, embedded in paraffin, and 5 μm sections were stained with hematoxylin and eosin (H&E). H&E stained tissues were microscopically analyzed to determine a colon histology score as described by Cooper et al. [15]. The maximum histology score was 10 based on the following scoring system: severity of inflammatory cell infiltration was scored 0: rare; 1: slightly dispersed cell infiltrate; 2: moderately increased cell infiltrates forming occasional cell foci; 3: severely large areas of cell infiltrates causing loss of tissue architecture. Extent of injury was scored 0: none; 1: mucosal; 2: mucosal and submucosal; 3: transmural. Crypt damage was scored 0: intact crypts; 1: basal on third damaged; 2: basal two third damaged; 3: only surface epithelium intact; 4: loss of entire crypt and epithelium. Rat anti-F4/80 (#MCA497B, AbD Serotec) or anti-FoxP3 (#14-5773-82, eBioscience) antibody (Ab) were used to visualize macrophages or regulatory T-cells with a goat anti-rat secondary Ab (#A11077, Invitrogen). Rabbit anti-CD11b (ab75476, Abcam), iNOS (ab3523, Abcam), and CD206 (ab64693, Abcam) Ab were used to detect myeloid cells, M1 and M2 macrophages, respectively; with alkaline phosphatase one-step polymer anti-mouse/rabbit/rat secondary Ab (Zytomed Systems) for visualization. Staining of hypoxia was performed by administration of pimonidazol (Hypoxyprobe^™^, HPI Inc.) prior to putting the mice to death; for detection the mouse anti-Hypoxyprobe-1 Ab (Hypoxyprobe^™^, HPI Inc.) in combination with M.O.M.^™^-Kit (Vector Labs) was used. HIF-1α or HIF-2α detection was achieved using rabbit anti-HIF-1α Ab (#10006421, Cayman Chemicals) or rabbit anti-HIF-2α Ab (#NB100-122, Novus) and the CSA-I Kit (DAKO).

### Statistical analyses

Statistical analyses were performed using the Graph Pad Prism 6.01 (USA) package software. Data-sets underwent Grubbs outlier test [18]. Differences among groups were assessed using One-way ANOVA or Two-way ANOVA with preselected groups or student’s t-test. Significance was set at P < 0.05. Data are presented as average values and mean standard error from experiments performed in triplicate (n = 3), except in vivo experiments (control groups n = 5, DSS-treated groups n = 6).

## Results

### Loss of myeloid HIF-1α does not affect tissue hypoxia during experimental colitis

**Fig 1:**
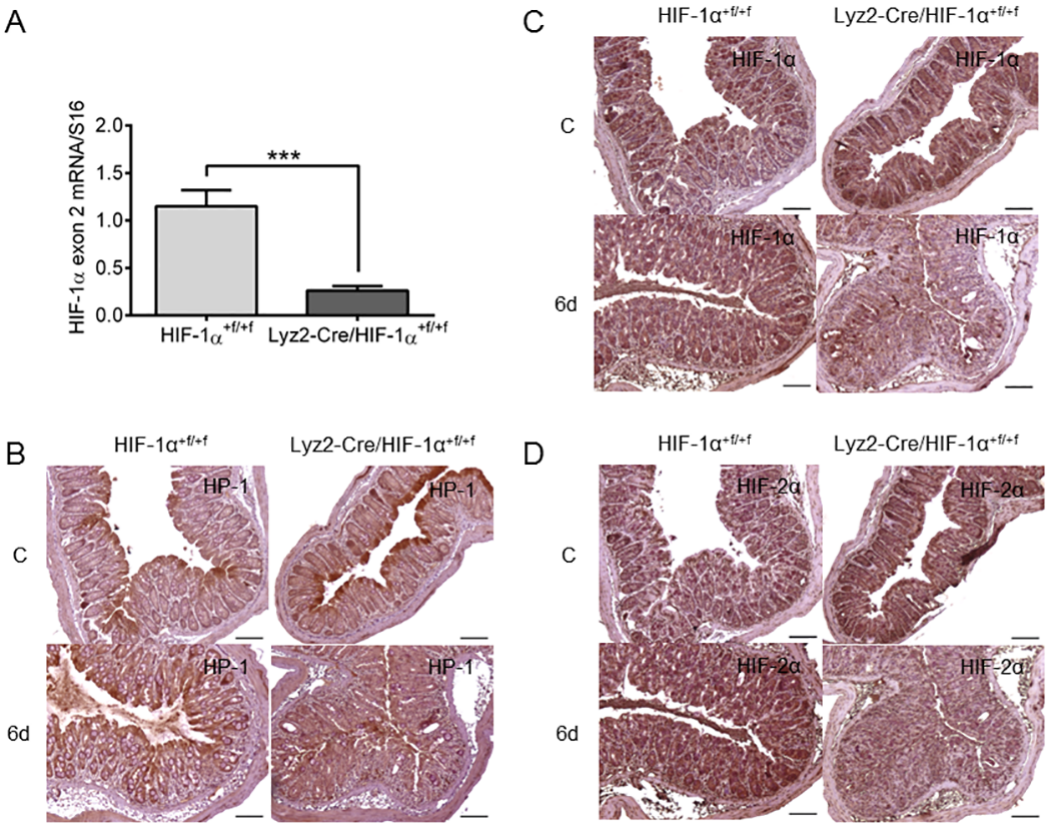
Hypoxia and HIF-1 accumulation after DSS-treatment. Real time PCR of HIF-1α exon 2 (**A**) in RNA samples of hypoxic treated BMDM of wild type (HIF-1α^+f/+f^) and knockout (Lyz2-Cre/HIF-1α^+f/+f^) mice with 14 samples/group. Each bar represents the mean value ± SEM. ***P < 0.001. Immunohistochemical staining of hypoxia with Hypoxyprobe-1 (HP-1) antibody (**B**) or staining of HIF-1α (**C**) and HIF-2α (**D**) in paraffin-embedded colon tissue of wild type (HIF-1α^+f/+f^) and knockout (Lyz2-Cre/HIF-1α^+f/+f^) mice after treatment with drinking water (C = control) or with 2.5% DSS for six days (6d). Note, that the HIF-1α antibody recognizes both wild type and knockout HIF-1α (lacking exon 2) and therefore detects (non-functional) HIF-1α protein in knockout mouse tissue. Representative images of stained colon slices of experiments with five or six mice/group. Original bars 100 μm.

To examine the role of myeloid HIF-1α during IBD we bred knockout mice with a cell-specific knockout of HIF-1α in myeloid cells (Lyz2-Cre/HIF-1α^+f/+f^) and their HIF-1α expressing wild type siblings (HIF-1α^+f/+f^). In isolated bone marrow derived macrophages knockout efficiency of HIF-1α exon 2 was about 80% on mRNA level (Fig 1A). Because the antibody recognizes the C-terminal region of HIF-1α, both wild type HIF-1α and HIF-1α knockout protein (lacking exon 2) are detected This sometimes leads to confusion because HIF-1α signals are detected in knockout mice (Fig 1C). However, since Exon 2 encodes for the DNA-binding domain knockout mice have no functional HIF-1α in cells of the myeloid lineage.

Hypoxia is known to play a role in IBD [19]. Karhausen et al. showed the enhancement of physiological hypoxia in colonic epithelium after induction of experimental colitis [11]. This was confirmed in the current study by Hypoxyprobe^™^-1 staining in combination with HIF-1α and HIF-2α detection in colon tissue after induction of experimental colitis (Fig 1B). Physiological hypoxia was observed in both control groups with extensive staining of the epithelial layer. In DSS-treated mice, however, staining for hypoxia extended throughout the whole mucosal layer of the colon. Corresponding to Hypoxyprobe-1 staining HIF-1α positive cells were found in the whole mucosal layer after DSS-treatment with more HIF-1α positive cells also in submucosal layers (Fig 1C and S1 Fig). HIF-2α expression in the colon was neither affected by treatment nor phenotype of mice and showed steady distribution throughout the colonic epithelium (Fig 1D).

### Loss of myeloid HIF-1α leads to a milder DSS-induced colitis

**Fig 2:**
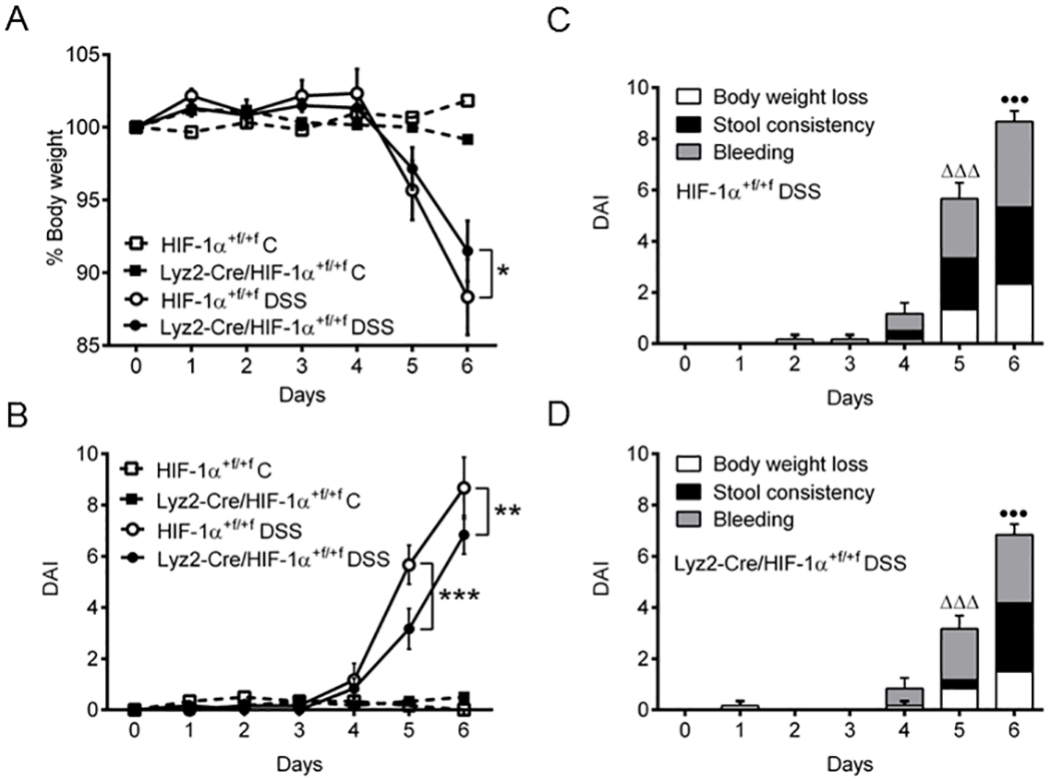
Effects of myeloid HIF-1α knockout on DSS-induced colitis. (**A**) Weight loss of original body weight and DAI (**B**) of wild type (HIF-1α^+f/+f^) and knockout (Lyz2-Cre/HIF-1α^+f/+f^) mice treated for six days (6d) with 2.5% DSS. Single values of DAI (body weight loss, stool consistency and rectal bleeding) are displayed for DSS-treated wild type (HIF-1α^+f/+f^) (**C**) and knockout (Lyz2-Cre/HIF-1α^+f/+f^) (**D**) mice. Data are representative for experiments with five or six mice/group. Each time point represents the mean value ± SEM. *P < 0.05; **P < 0.01 and ***P < 0.001 compared as indicated. • displays significance in body weight loss and Δ displays significance in stool consistency at respective days between (C) and (D).

**Fig 3:**
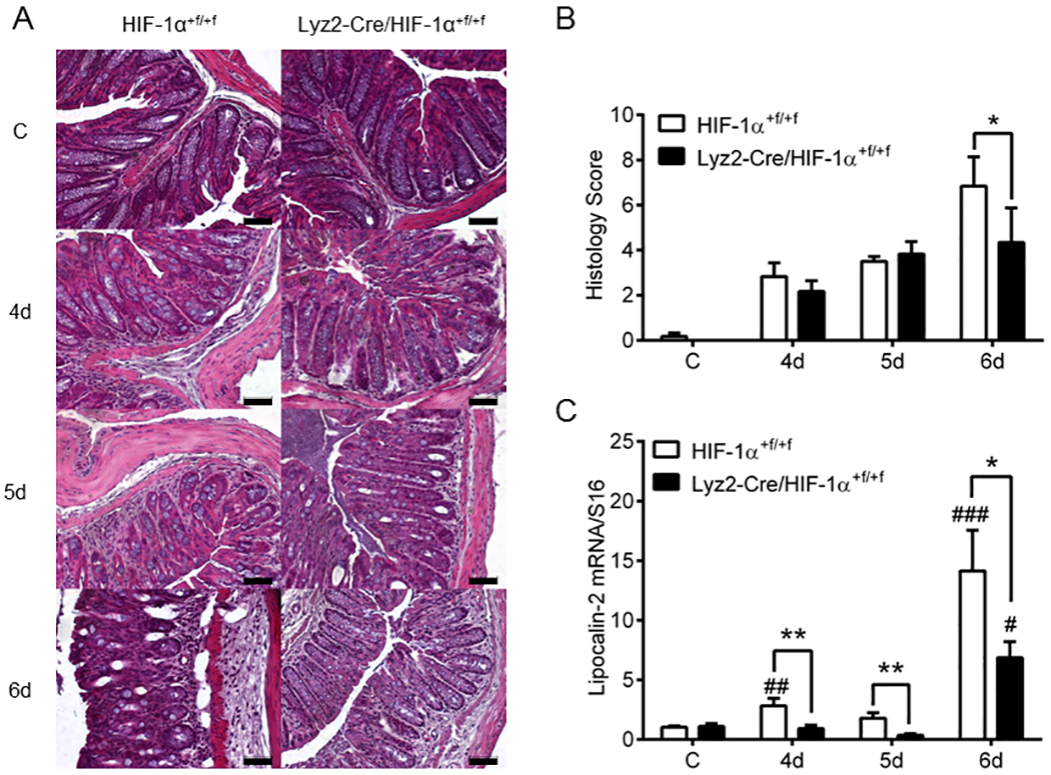
Colon tissue damage in DSS-induced colitis in mice lacking myeloid HIF-1α. H&E staining (**A**) and histology scoring (**B**) of paraffin-embedded colon sections from wild type (HIF-1α^+f/+f^) and knockout (Lyz2-Cre/HIF-1α^+f/+f^) mice treated for four to six days (4d-6d) with 2.5% DSS. (**C**) Real time PCR of Lipocalin-2 in colon RNA samples of wild type (HIF-1α^+f/+f^) and knockout (Lyz2-Cre/HIF-1α^+f/+f^) mice treated as in (A). Data are representative for experiments with five or six mice/group. Each time point represents the mean value ± SEM. *P < 0.05; **P < 0.01 and ***P < 0.001 compared as indicated. # displays significance to respective control. Original bars, 50 μm.

Wild type and knockout mice were challenged in a DSS-induced model of colitis with or without administration of 2.5% DSS via drinking water. Control animals did not lose weight (Fig 2A) and had no signs of rectal bleeding or diarrhea at any time point of the experiment (Fig 2B). After four days of DSS-treatment both DSS-treated groups started to lose weight (Fig 2A) and showed first signs of rectal bleeding and diarrhea which resulted in an increasing DAI. After six days of DSS-treatment wild type mice exhibited stronger colitis with weight loss of about 12% of their original weight and DAIs of 8, whereas knockout mice lost significantly less weight and had a lower DAI (Fig 2B). Among the single parameters used for calculation of the DAI especially weight loss and stool consistency were milder in DSS-treated knockout mice, whereas bleeding was comparable between the groups (Fig 2C and 2D). Administration of DSS resulted in similar shortening of colon length in both knockout and wild type mice without any significant difference between the two groups (S2 Fig). Colonic damage was observed in H&E stained tissue samples (Fig 3A) and quantified with a histology score (Fig 3B). According to weight loss and DAI, first damage was observed after four days of DSS-treatment in both groups. Differences on histology were apparent after six days of DSS-treatment when colon tissue of wild type mice was damaged to a greater extent. Additionally, Lipocalin-2, a marker for gut inflammation in stool, serum, and colon samples during DSS-experiments [20], was expressed in whole colon RNA of wild type mice at day four but significantly higher at day six of DSS-treatment when compared to knockout mice (Fig 3C).

### Loss of myeloid HIF-1α reduces immune cell recruitment to inflammatory sites

**Fig 4:**
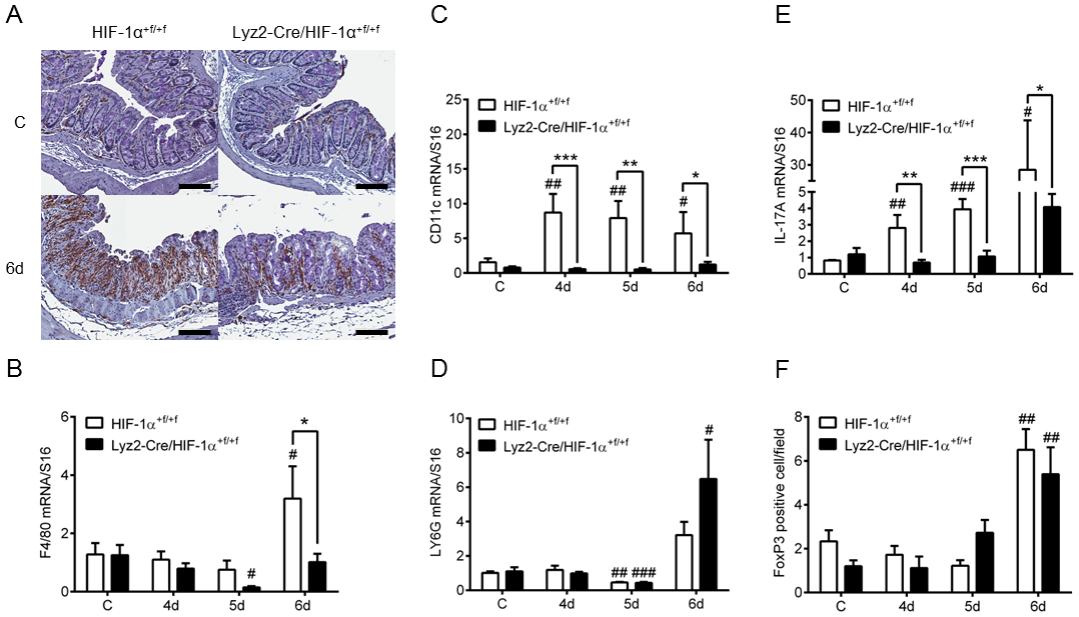
Accumulation of macrophages in colon tissue of mice lacking myeloid HIF-1α after DSS-treatment. (**A**) F4/80 staining of paraffin-embedded colon sections from wild type (HIF-1α^+f/+f^) and knockout (Lyz2-Cre/HIF-1α^+f/+f^) mice treated with drinking water (C) or with 2.5% DSS (6d) for six days (6d). Real time PCR of markers for macrophages (F4/80) (**B**), dendritic cells (CD11c) (**C**), neutrophils (LY6G) (**D**) and Th17 cells (IL-17A) (**E**) in colon RNA samples of wild type (HIF-1α^+f/+f^) and knockout (Lyz2-Cre/HIF-1α^+f/+f^) mice treated for four to six days (4d-6d) with 2.5% DSS. (**F**) Number of FoxP3 positive cells/field of view in 4 fields/colon section of wild type (HIF-1α^+f/+f^) and knockout (Lyz2-Cre/HIF-1α^+f/+f^) mice treated as in (B). Data are representative for experiments with five or six mice/group. Each time point represents the mean value ± SEM. *P < 0.05; **P < 0.01 and ***P < 0.001 compared as indicated. # displays significance to respective control. Original bars, 100 μm.

Knockout of HIF-1α with Lyz2-Cre recombinase mainly affects neutrophils and macrophages [12]. The presence of macrophages in colon tissue during DSS-induced colitis was observed via histology. F4/80 staining was sporadic in colon tissue of untreated but strong in colon tissue of DSS-treated mice. DSS-treated wild type mice revealed more pronounced F4/80 staining when compared to knockout mice (Fig 4A and S3 Fig). Corresponding to this result F4/80 mRNA expression was markedly increased at day six of DSS-treatment solely in wild type mice (Fig 4B). mRNA expression levels of CD11c (Fig 4C)—amongst others a marker for dendritic cells (DCs)- and the Th17 cell marker IL-17A (Fig 4E) were increased at all time points of DSS-treatment only in wild type mice. mRNA expression level of LY6G, a marker for neutrophils, was increased after six days of DSS-treatment in both groups, but not different between wild type and knockout mice (Fig 4D).

**Fig 5:**
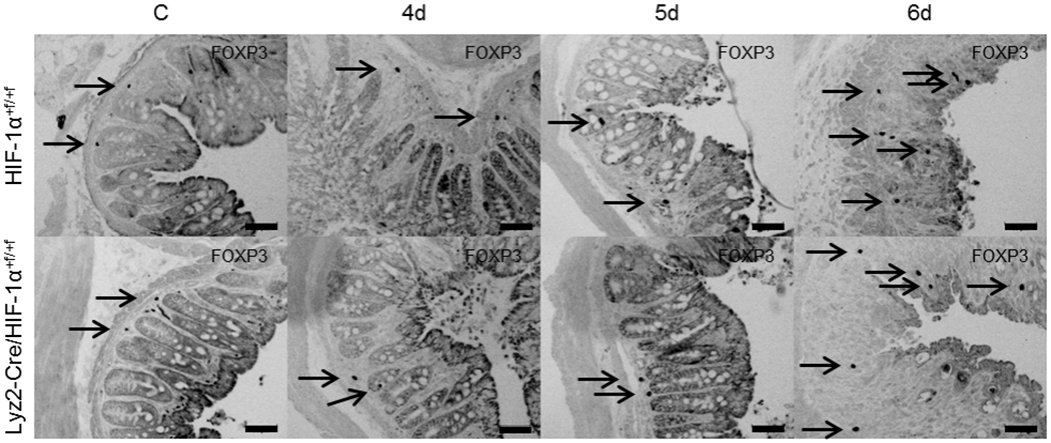
Accumulation of regulatory T cells in colon tissue after DSS-treatment. FoxP3 staining of paraffin-embedded colon sections from wild type (HIF-1α^+f/+f^) and knockout (Lyz2-Cre/HIF-1α^+f/+f^) mice treated for four to six days (4d-6d) with 2.5% DSS. FoxP3 positive staining is indicated by arrows. Data are representative for experiments with five or six mice/group. Original bars, 50 μm.

The presence of regulatory T cells was evaluated by FoxP3 staining (Fig 5). Quantitative analysis of FoxP3 staining demonstrated equal numbers of regulatory T cells after six days of DSS-treatment in both groups (Fig 4F).

### Myeloid HIF-1α plays a role for inflammatory cytokine mRNA expression levels in colon tissue

**Fig 6:**
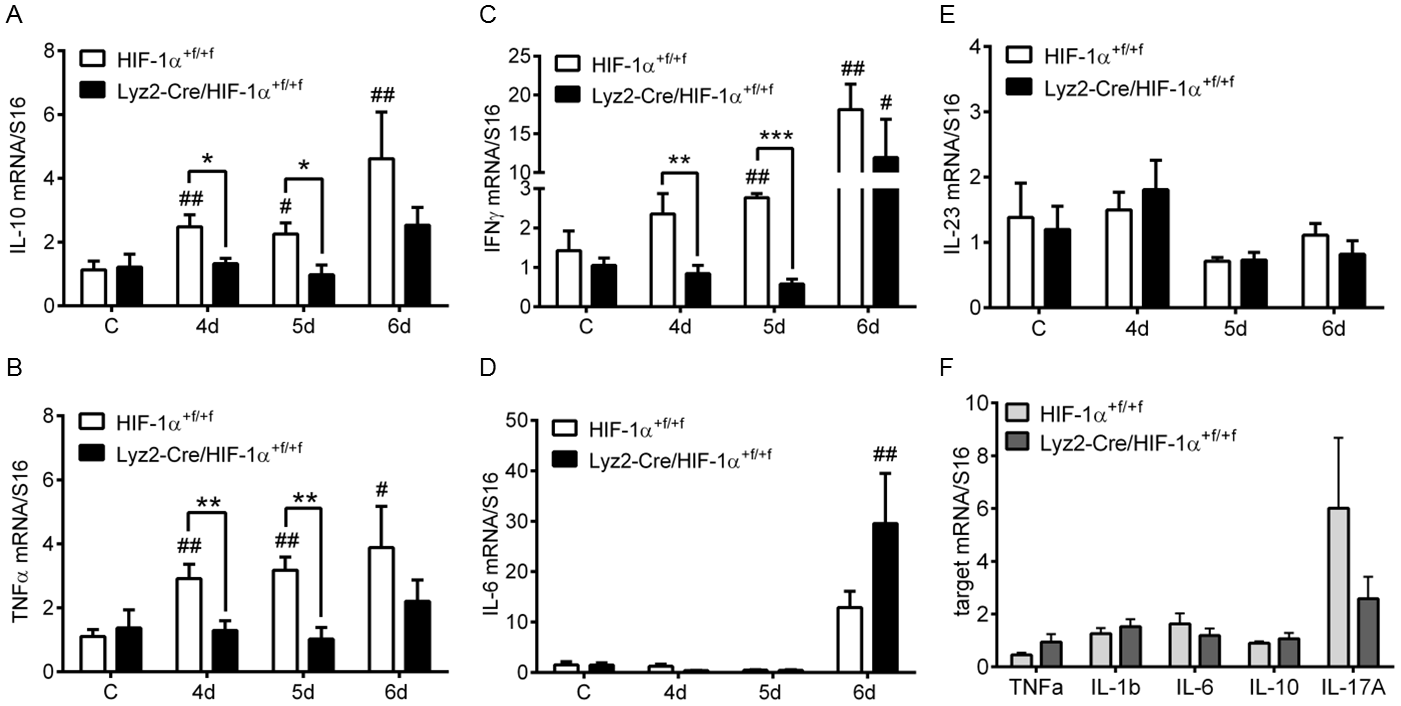
Expression of cytokine mRNA during DSS-treatment in mice without myeloid HIF-1α. Real time PCR of IL-10 (**A**), TNFα (**B**), IFNγ (**C**), IL-6 (**D**) and IL-23 (**E**) in colon RNA samples of wild type (HIF-1α^+f/+f^) and knockout (Lyz2-Cre/HIF-1α^+f/+f^) mice treated for four to six days (4d-6d) with 2.5% DSS. Data are representative for experiments with five or six mice/group. (**F**) Real time PCR of TNFα, IL-1ß, IL-6, IL-10, and IL-17A in RNA samples of bone marrow derived macrophages from wild type (HIF-1α^+f/+f^) and knockout (Lyz2-Cre/HIF-1α^+f/+f^) mice treated for six hours with 1% O_2_. Each time point represents the mean value ± SEM. *P < 0.05; **P < 0.01 and ***P < 0.001 compared as indicated. # displays significance to respective control.

To investigate if reduced expression levels of immune cell markers were accompanied by diminished cytokine mRNA expression levels, a number of inflammatory bowel disease related cytokines were analyzed. mRNA expression levels of cytokines like IL-10 (Fig 6A), TNFα (Fig 6B), and IFNγ (Fig 6C) continuously increased over time in colons of DSS-treated wild type mice. Knockout mice showed no increase in IL-10, IFNγ, and TNFα mRNA expression before day 6 of DSS-treatment while wild type mice started to increase cytokine expression earlier. This resulted in significant differences between wildtype and knockout mice on days 4 and 5 for these three cytokines. IL-6 mRNA expression levels were markedly increased after six days of DSS-treatment in both DSS-treated groups but were higher in knockout mice (Fig 6D). IL-23 mRNA expression showed no alteration during induction of colonic inflammation (Fig 6E). To clarify whether reduced cytokine mRNA expression levels were directly related to the loss of HIF-1α, bone marrow derived macrophages where cultivated from knockout and wild type mice and challenged for six hours under hypoxic conditions. mRNA expression levels of the cytokines TNFα, IL-1β, IL-6 and IL-10 (Fig 6F) showed an equal increase between bone marrow derived macrophages of wild type and knockout mice. Only mRNA expression levels of IL-17A were higher in bone marrow derived macrophages of wild type mice when compared to knockout mice (Fig 6F). To investigate whether neutrophils contribute to cytokine expression, neutrophils were isolated from wild type and knockout mice, challenged with hypoxia and cytokine mRNA expression was analyzed. Although neutrophils also had a knockout efficiency of HIF-1α exon 2 of about 80% (S4 Fig), there was no effect of knockout on mRNA expression levels of cytokines like TNFα, IL-1β, IL-6, IL-10 and IL-17A (S4 Fig).

**Fig 7:**
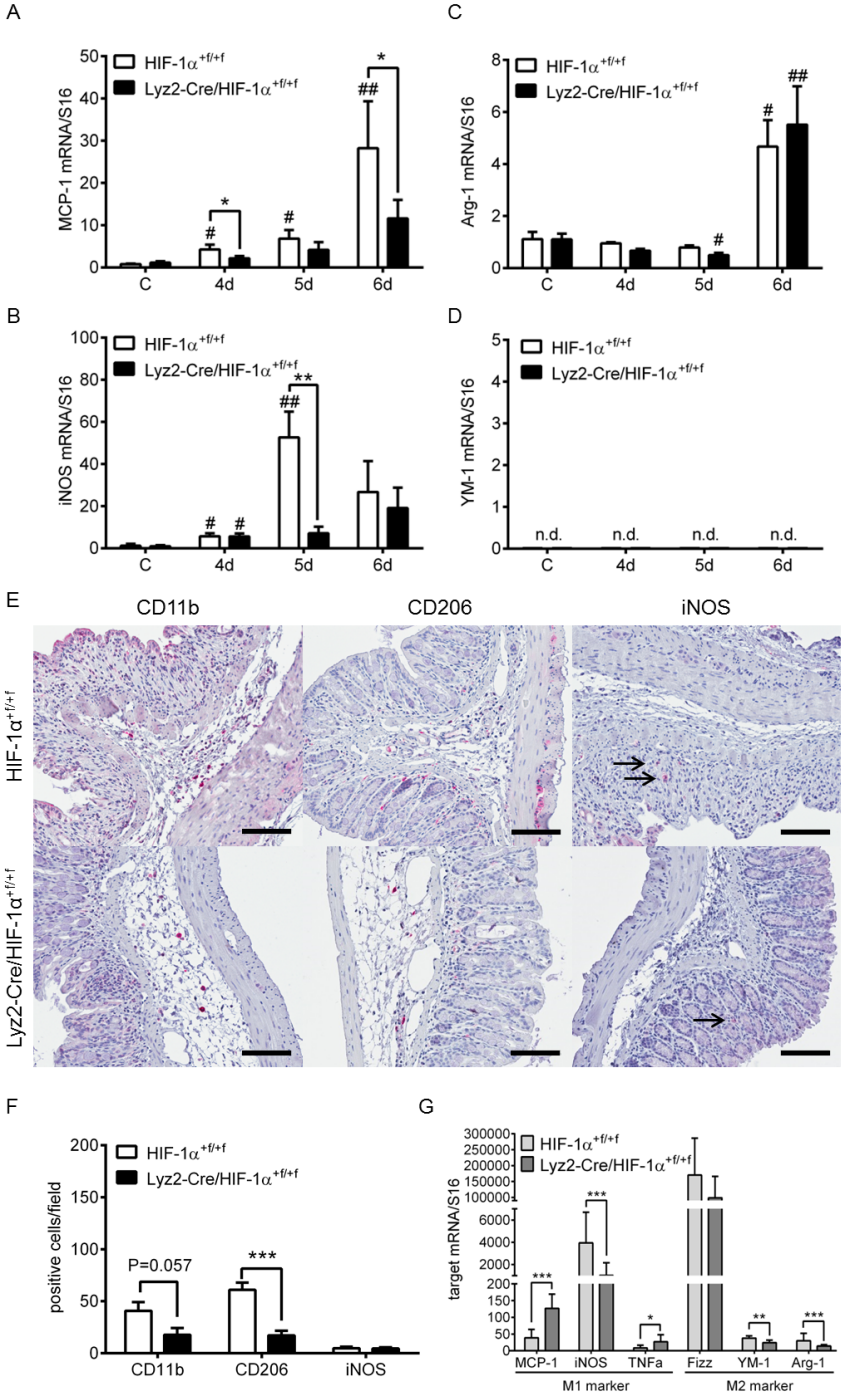
Macrophage polarization in the inflamed colon or in bone marrow derived macrophage cultures of mice with and without myeloid HIF-1α. Real time PCR of MCP-1 (**A**), iNOS (**B**), Arginase-1 (Arg-1) (**C**) and YM-1 (**D**) in RNA samples of wild type (HIF-1α^+f/+f^) and knockout (Lyz2-Cre/HIF-1α^+f/+f^) mice treated for four to six days (4d-6d) with 2.5% DSS. (**E**) Staining of myeloid cells (CD11b), M1 (iNOS) and M2 (CD206) macrophages of paraffin-embedded colon sections from wild type (HIF-1α^+f/+f^) and knockout (Lyz2-Cre/HIF-1α^+f/+f^) mice treated for six days with 2.5% DSS. iNOS positive staining is indicated by arrows. Original bars, 100 μm. Data are representative for experiments with six mice/group. (**F**) Numbers of CD11b, CD206 and iNOS positive cells/field of view in colon sections of wild type (HIF-1α^+f/+f^) and knockout (Lyz2-Cre/HIF-1α^+f/+f^) mice treated as in (E). (**G**) Real time PCR of MCP-1, iNOS, TNFα, Fizz, YM-1 and Arginas-1 in RNA samples of M1/M2 polarized bone marrow derived macrophages from wild type (HIF-1α^+f/+f^) and knockout (Lyz2-Cre/HIF-1α^+f/+f^) mice. Each time point represents the mean value ± SEM. *P < 0.05; **P < 0.01 and ***P < 0.001 compared as indicated. # displays significance to respective control; n.d. = not detectable.

Takeda et al. showed HIF-1 and HIF-2 to play a role in regulating M1/M2 polarization with HIF-1 regulating *inos* expression and the M1 state and HIF-2 *arginase-1* expression and the M2 state [21]. To investigate if loss of myeloid HIF-1α affected M1/M2 polarization, M1 and M2 markers were stained in colon tissue of DSS-treated mice. In addition, mRNA expression of M1 and M2 markers was examined in whole colon RNA and M1/M2 polarized bone marrow derived macrophages. mRNA expression of M1 markers MCP-1 (Fig 7A) and iNOS (Fig 7B) showed an induction after DSS-treatment in wild type mice but only mildly in knockout mice. Expression of the M2 marker Arginase-1 was increased after 6 days of DSS-treatment in both wild type and knockout mice (Fig 7C), whereas the M2 marker YM-1 was not detectable in whole colon RNA at all (Fig 7D). In colon tissue of DSS-treated wild type mice significantly more CD11b and CD206 positive cells were found than in knockout mice, whereas iNOS staining was nearly undetectable in both groups (Fig 7E and 7F). Additionally, CD11b staining was also more pronounced in wild type mice early on day 4 of DSS-treatment (S5 Fig). If directly investigated in M1/M2 polarized bone marrow derived macrophages, mRNA expression of M1 markers MCP-1 and TNFα (Fig 7G) were significantly increased in M1 polarized bone marrow derived macrophages isolated from knockout mice. On the other hand, the M2 markers YM-1 and Arg-1 (Fig 7G) were markedly decreased in M2 stimulated bone marrow derived macrophages from knockout mice.

## Discussion

IBD, comprising Crohn’s disease and ulcerative colitis, are chronic, relapsing intestinal inflammations with continually increasing incidence. Although our current understanding of the pathogenesis of IBD proceeds, further research is needed to fully elucidate the complex interplay between genetics, immune response, and alterations of the microbiome causing IBD [22].

Previous studies revealed a protective role of HIF-1 in immune cells like DC and T cells, and epithelial cells during IBD [9–11]. In these studies the loss of HIF-1α resulted in a more severe inflammation due to impaired immune cell balance or disturbed epithelial barrier. For myeloid cells Cramer et al. showed a role for HIF-1α in cell aggregation, motility and invasiveness, and—with respect to cell function—for bacterial killing [6]. Mice lacking myeloid HIF-1α had a diminished inflammatory response in a model of acute skin inflammation. Here, the loss of myeloid HIF-1α resulted in a decreased inflammatory cell infiltration, which prompted Cramer et al. to postulate a “critical role of HIF-1 in infiltration at the very earliest stage of inflammatory cell recruitment” [6]. Also Scheerer at al. showed a delayed invasion of macrophages lacking HIF-1α to skeletal muscle injury sites caused by mechanical trauma [23].

To address the effects of myeloid HIF-1α loss on experimental colon inflammation DSS-induced colitis was examined in mice with functional HIF-1α and mice with deficient HIF-1α function restricted to myeloid cells. Mice with loss of myeloid HIF-1α showed an overall milder disease outcome of DSS-induced colitis (Fig 2). This could well result from diminished motility and invasiveness of myeloid cells lacking HIF-1α (S5 Fig), causing reduced recruitment of additional inflammatory immune cells like DC and Th17 cells (Fig 4). Comparable results were achieved by Marshall et al. [24] through administration of an anti-colony stimulating factor-1 (CSF-1) antibody during DSS-induced colitis. CSF-1 serves as primary mediator of macrophage differentiation, proliferation, activation, and migration. Inhibition of CSF-1 suppressed DSS-induced colitis due to diminished immune cell infiltration of T cells and macrophages in colon tissue and reduction of TNFα, IL-1β and IL-6 levels [24]. Likewise, in the present study we found reduced expression of various IBD-related inflammatory cytokines like TNFα and IFNγ in mice lacking myeloid HIF-1α. Additionally, mRNA expression of IL-10 which showed no alteration in the work of Marshall et al. was reduced in myeloid HIF-1α deficient mice (Fig 6). This cytokine pattern may originate in diminished numbers of inflammatory immune cell accumulation in mice lacking myeloid HIF-1α.

Macrophage polarization has been linked to altered HIF mRNA expression with mainly HIF-1α in M1 macrophages and HIF-2α in M2 macrophages [21]. In this study, loss of myeloid HIF-1α resulted in a reduced M2 phenotype in colon tissue of DSS-treated mice, but no clear effects on the M1 phenotype. MCP-1 and TNFα mRNA expression was significantly increased in bone marrow derived macrophages lacking HIF-1α, whereas YM-1, Arginase-1 and iNOS expression was clearly decreased. Significantly lower iNOS mRNA expression in whole colon RNA was also observed in mice lacking HIF-1α on day 5 when the highest difference in DAI between wild type and knockout was seen. However, iNOS mRNA levels were increased to similar levels on day 6 when we also saw no difference in iNOS protein positive cells between the mice (Fig 7). Collectively, myeloid HIF-1α knockout affects markers of M1 and M2 polarization but does not result in a clear reduction of the M1 phenotype.

Previously, numbers of Th17 cells were reported to be exclusively increased in patients with IBD when compared to healthy controls or patients with infectious or ischemic colitis. It was postulated that T cells, monocytes, and macrophages are the main source of IL-17 [25]. Dang et al. demonstrated a pivotal role of HIF-1 for Th17 cell development through direct activation of IL-17 transcription [26]. Furthermore, Marks et al. revealed HIF-1α to regulate IL-12p40 expression with HIF-stabilization resulting in induction of IL-12p40 which was accompanied by diminished Th1 and Th17 cell response in mucosal inflammation [27]. It is also known that IL-17A influences functions of monocytes and macrophages and that the inhibition of IL-17A prevents atherosclerotic lesion progression—another chronic inflammatory disease—in mice and human through reducing inflammatory burden and cellular infiltration [28]. Mice deficient in myeloid HIF-1α showed reduced expression of IL-17 in our DSS-induced colitis model (Fig 4E). This may originate from the lower number of macrophages in colon tissue of these mice, resulting in less recruitment of Th17 cells. On the other hand, loss of functional HIF-1α in monocytes and macrophages led to reduced IL-17A expression although this was not significant (Fig 6F). Moreover, IL-23 which is mainly secreted by resident monocytes stimulates innate lymphoid cells to secrete IL-17 [29] and loss of IL-23 receptor led to lower IL-17A expression during DSS-induced colitis [30]. Herein, we found no obvious differences in IL-23 expression between the two groups of DSS-treated mice (Fig 6E). It is possible that we missed peak IL-23 expression because we could already detect elevated IL-17A mRNA levels in the colon of DSS-treated WT mice. Changes in overall IL-23 mRNA would be expected prior to the IL-17 response. Comparable results have been shown in experimental colitis with deletion of HIF-1α in dendritic cells [9] and T-cells [10].

Regulatory T cells (Treg) maintain the well-balanced immune response especially in the gut. Treg are characterized by expression of the transcription factor FoxP3 which was shown to be regulated by HIF-1α [26,31]. We recently reported the important role of dendritic HIF-1α for the sufficient induction of Treg during intestinal inflammation [9]. In the present study—addressing myeloid HIF-1α—DSS-treatment led to an equal increase in FoxP3 positive cells in both groups of mice (Figs 4F and 5), indicating a minor role of myeloid HIF-1α for the induction of FoxP3 and therefore for maintaining sufficient numbers of Treg.

Myeloid cells like neutrophils and macrophages are the first line of defense in case of infection. Release of various chemokines like TNFα leads to recruitment of further immune cells like DC [32]. Diminished expression levels of cytokines such as TNFα were detected in colon tissues of mice lacking myeloid HIF-1α, which are likely caused by the reduced number of macrophages. This subsequently led to reduced recruitment of inflammatory immune cells like DC and Th17 cells.

As mentioned before, HIF-1 deficiency in epithelial cells and immune cells like DCs and T cells led to an increased inflammation in experimental colitis. Hence, therapeutical administration of hydroxylase inhibitors and therefore an overall stabilization of HIF during experimental colitis was performed and showed protective effects [33]. To reduce potential side effects due to systemic stabilization of all HIF-isoforms like increased hematocrit or tumor development, Tambuwala et al. established targeted delivery of a hydroxylase inhibitor with lower dose which avoids systemic hydroxylase inhibition [34]. Since macrophages represent the most abundant leukocytes in the intestinal lamina propria especially in the colon [35] these immune cells will play an important role regarding the treatment of IBD and might be affected by non-specific HIF-1 stabilization. Of note, it was shown that increased HIF-1 expression in myeloid cells led to a hyperinflammatory response [6]. Thus, cell specific targeting of HIF-1 stabilization or inhibition as Tambuwala and coworkers have established needs to be further developed.

Using mice with a conditional knockout of HIF-1α in myeloid cells in a DSS-induced model of colitis we report a disease promoting role of myeloid HIF-1 during intestinal inflammation. This is in contrast to the inflammation-dampening role of HIF-1α in dendritic cells in the same colitis model (9). Our findings provide evidence for antagonistic roles of HIF-1 in immune cells during inflammatory bowel disease depending on the cell-specific context.

## Supporting information

S1 Fig Hypoxia after DSS-treatment. Immunohistochemical staining of hypoxia with Hypoxyprobe-1 (HP-1) antibody in paraffin-embedded colon tissue of wild type (HIF-1α^+f/+f^) and knockout (Lyz2-Cre/HIF-1α^+f/+f^) mice after treatment with drinking water (C = control) or with 2.5% DSS for six days (6d). Overview of representative DAB image. Hematoxylin and DAB channels were separated using the Colour Deconvolution plugin of ImageJ. Original bars 100 μm.
(TIF)

S2 Fig Colon shortening after DSS-induced colitis. Colon length was measured directly after removal of wild type (HIF-1α^+f/+f^) and knockout (Lyz2-Cre/HIF-1α^+f/+f^) mice treated for four to six days (4d-6d) with 2.5% DSS. Data are representative for experiments with five or six mice/group. Each time point represents the mean value ± SEM.
(TIF)

S3 Fig Reduced accumulation of macrophages in colon tissue of mice lacking myeloid HIF-1α after DSS-treatment. F4/80 staining of paraffin-embedded colon sections from wild type (HIF-1α^+f/+f^) and knockout (Lyz2-Cre/HIF-1α^+f/+f^) mice treated with 2.5% DSS (6d) for six days (6d). Overview of representative DAB image. Hematoxylin and DAB channels were separated using the Colour Deconvolution plugin of ImageJ. Original bars, 100 μm.
(TIF)

S4 Fig Knockout efficiency and cytokine expression in neutrophils. Real time PCR of HIF-1α exon 2 (**A**) and TNFα, IL-1ß, IL-6, IL-10, and IL-17A (**B**) in RNA samples of isolated neutrophils from wild type (HIF-1α^+f/+f^) and knockout (Lyz2-Cre/HIF-1α^+f/+f^) mice treated for three hours with 1% O_2_. Each time point represents the mean value ± SEM.
(TIF)

S5 Fig Myeloid cells in the inflamed colon. (**A**) Staining of myeloid cells (CD11b) of paraffin-embedded colon sections from wild type (HIF-1α^+f/+f^) and knockout (Lyz2-Cre/HIF-1α^+f/+f^) mice treated for four (4d) and five (5d) days with 2.5% DSS. Original bars, 100 μm. Data are representative for experiments with six mice/group. (**B**) Numbers of CD11b positive cells/field of view in colon sections of wild type (HIF-1α^+f/+f^) and knockout (Lyz2-Cre/HIF-1α^+f/+f^) mice treated as in (A). Each time point represents the mean value ± SEM. *P < 0.05; compared as indicated.
(TIF)
