# Supplementary figures and images for: Knockdown of myeloid cell hypoxia-inducible factor-1α ameliorates the acute pathology in DSS-induced colitis
Source: PLoS One. 2017 Dec 20;12(12):e0190074. doi: 10.1371/journal.pone.0190074 (PMC5738114; doi:10.1371/journal.pone.0190074)

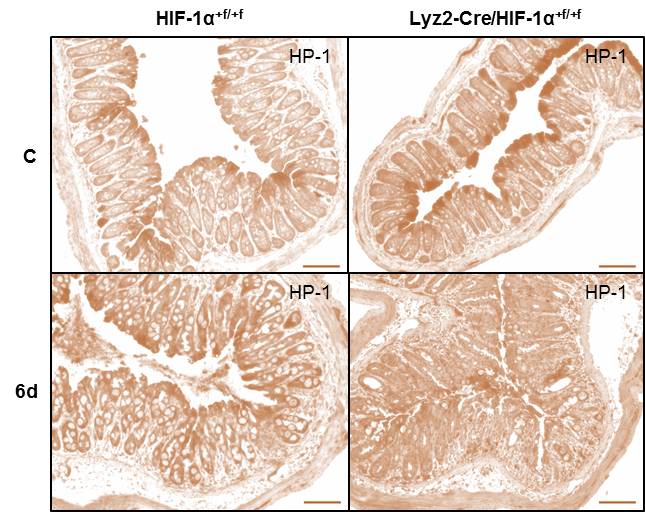

Supplement: S1 Fig — Immunohistochemical staining of hypoxia with Hypoxyprobe-1 (HP-1) antibody in paraffin-embedded colon tissue of wild type (HIF-1α+f/+f) and knockout (Lyz2-Cre/HIF-1α+f/+f) mice after treatment with drinking water (C = control) or with 2.5% DSS for six days (6d). Overview of representative DAB image. Hematoxylin and DAB channels were separated using the Colour Deconvolution plugin of ImageJ. Original bars 100 μm. (TIF) [file pone.0190074.s001.tif]

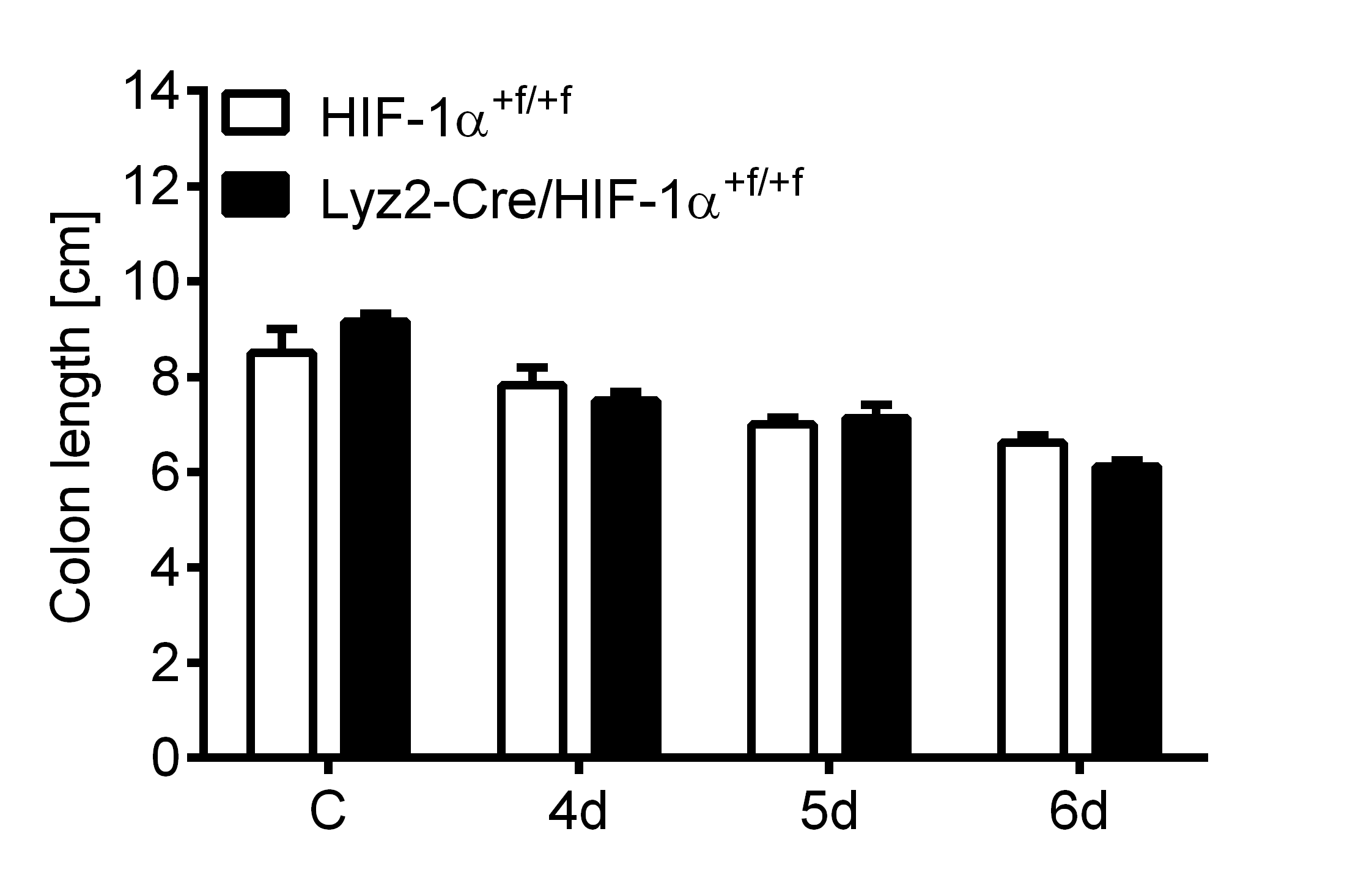

Supplement: S2 Fig — Colon length was measured directly after removal of wild type (HIF-1α+f/+f) and knockout (Lyz2-Cre/HIF-1α+f/+f) mice treated for four to six days (4d-6d) with 2.5% DSS. Data are representative for experiments with five or six mice/group. Each time point represents the mean value ± SEM. (TIF) [file pone.0190074.s002.tif]

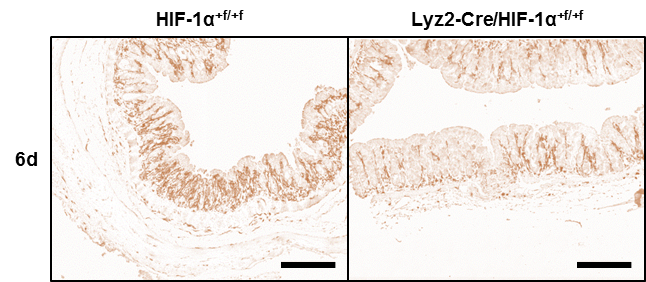

Supplement: S3 Fig — F4/80 staining of paraffin-embedded colon sections from wild type (HIF-1α+f/+f) and knockout (Lyz2-Cre/HIF-1α+f/+f) mice treated with 2.5% DSS (6d) for six days (6d). Overview of representative DAB image. Hematoxylin and DAB channels were separated using the Colour Deconvolution plugin of ImageJ. Original bars, 100 μm. (TIF) [file pone.0190074.s003.tif]

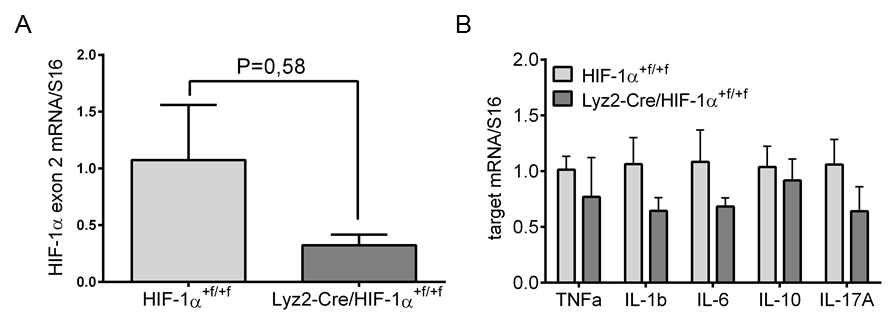

Supplement: S4 Fig — Real time PCR of HIF-1α exon 2 (A) and TNFα, IL-1ß, IL-6, IL-10, and IL-17A (B) in RNA samples of isolated neutrophils from wild type (HIF-1α+f/+f) and knockout (Lyz2-Cre/HIF-1α+f/+f) mice treated for three hours with 1% O2. Each time point represents the mean value ± SEM. (TIF) [file pone.0190074.s004.tif]

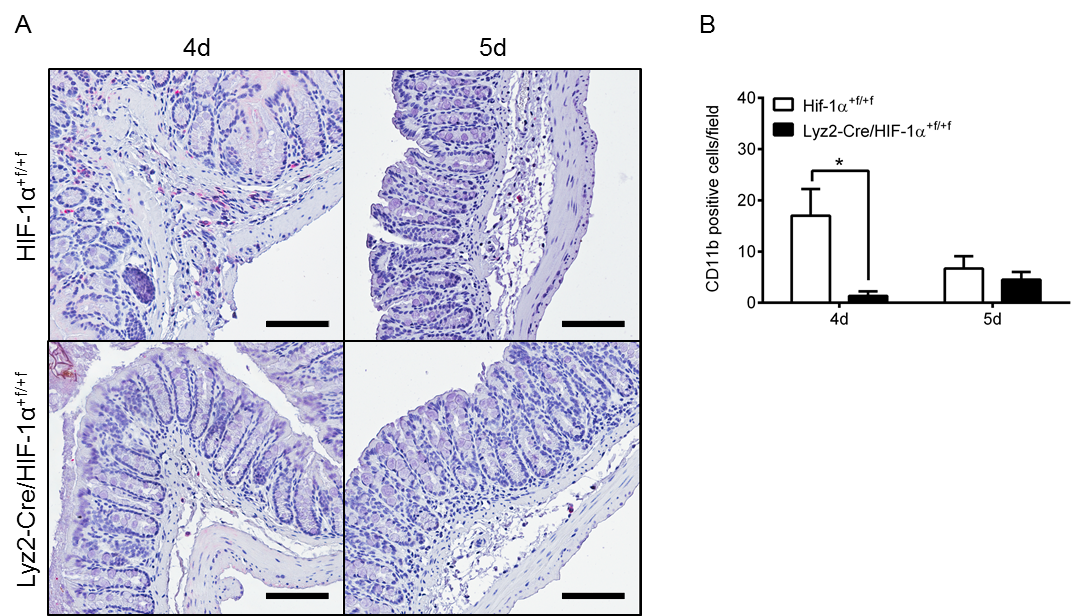

Supplement: S5 Fig — (A) Staining of myeloid cells (CD11b) of paraffin-embedded colon sections from wild type (HIF-1α+f/+f) and knockout (Lyz2-Cre/HIF-1α+f/+f) mice treated for four (4d) and five (5d) days with 2.5% DSS. Original bars, 100 μm. Data are representative for experiments with six mice/group. (B) Numbers of CD11b positive cells/field of view in colon sections of wild type (HIF-1α+f/+f) and knockout (Lyz2-Cre/HIF-1α+f/+f) mice treated as in (A). Each time point represents the mean value ± SEM. *P < 0.05; compared as indicated. (TIF) [file pone.0190074.s005.tif]
